# Reduction in Methane Emissions From Acidified Dairy Slurry Is Related to Inhibition of *Methanosarcina* Species

**DOI:** 10.3389/fmicb.2018.02806

**Published:** 2018-11-20

**Authors:** Jemaneh Habtewold, Robert Gordon, Vera Sokolov, Andrew VanderZaag, Claudia Wagner-Riddle, Kari Dunfield

**Affiliations:** ^1^School of Environmental Sciences, University of Guelph, Guelph, ON, Canada; ^2^Department of Geography and Environmental Studies, Wilfrid Laurier University, Waterloo, ON, Canada; ^3^Agriculture and Agri-Food Canada, Ottawa, ON, Canada

**Keywords:** dairy manure, greenhouse gas, manure acidification, methane, methanogens

## Abstract

Liquid dairy manure treated with sulfuric acid was stored in duplicate pilot-scale storage tanks for 120 days with continuous monitoring of CH_4_ emissions and concurrent examination of changes in the structure of bacterial and methanogenic communities. Methane emissions were monitored at the site using laser-based Trace Gas Analyzer whereas quantitative real-time polymerase chain reaction and massively parallel sequencing were employed to study bacterial and methanogenic communities using 16S rRNA and methyl-coenzyme M Reductase A (*mcrA*) genes/transcripts, respectively. When compared with untreated slurries, acidification resulted in 69–84% reductions of cumulative CH_4_ emissions. The abundance, activity, and proportion of bacterial communities did not vary with manure acidification. However, the abundance and activity of methanogens (as estimated from *mcrA* gene and transcript copies, respectively) in acidified slurries were reduced by 6 and 20%, respectively. Up to 21% reduction in *mcrA* transcript/gene ratios were also detected in acidified slurries. Regardless of treatment, *Methanocorpusculum* predominated archaeal 16S rRNA and *mcrA* gene and transcript libraries. The proportion of *Methanosarcina*, which is the most metabolically-diverse methanogen, was the significant discriminant feature between acidified and untreated slurries. In acidified slurries, the relative proportions of *Methanosarcina* were ≤ 10%, whereas in untreated slurries, it represented up to 24 and 53% of the *mcrA* gene and transcript libraries, respectively. The low proportions of *Methanosarcina* in acidified slurries coincided with the reductions in CH_4_ emissions. The results suggest that reduction of CH_4_ missions achieved by acidification was due to an inhibition of the growth and activity of *Methanosarcina* species.

## Introduction

Livestock production is a significant source of methane (CH_4_) emissions (e.g., 119.1 ± 18.2 Tg in 2011) (Wolf et al., 2017), mainly from enteric fermentation and manure management of dairy farming operations (Laubach et al., 2015; Jayasundara et al., 2016; Wolf et al., 2017). The large volumes of manure produced annually from intensive dairy farming operations areusually stored in slurry form (VanderZaag et al., 2013), which create environments conducive to CH_4_ production (Grant et al., 2015; Petersen, 2018). To reduce CH_4_ emissions from such storage systems, strategies such as reduction of aged manure (inoculants), crust development for potential aerobic CH_4_ oxidation, and manure acidification using sulfuric acid (H_2_O_4_) have been reported (Petersen et al., 2012; Sommer et al., 2017; Habtewold et al., 2018). Sulfuric acid-based acidification of liquid dairy manure has primarily been used to abate ammonia (NH_3_) emissions, but can also reduce CH_4_ emissions (Ottosen et al., 2009; Petersen et al., 2012; Fangueiro et al., 2015; Sommer et al., 2017). For instance, CH_4_ emissions from cattle slurry were reduced by 68% by acidification to pH 5.5 with H_2_SO_4_ (Sommer et al., 2017). More than 90% reduction of CH_4_ emissions from acidified pig slurry were also reported by Petersen et al. (2014). In fact, acidification of stored liquid dairy manure has already been implemented at farm-scale in some countries such as Denmark. In slurries, H_2_SO_4_ itself is expected to be converted to plant-available sulfate sulfur (Eriksen et al., 2008), and H_2_SO_4_ would not be found in the slurry after acidification has already occurred. However, there are no data available about the effects of manure acidification on the activities of microbial communities in stored liquid dairy manure.

In stored liquid dairy manure, complete degradation of complex organic matter involves different groups of microbial communities (hydrolytic, acidogenic, acetogenic, and methanogenic). The pH range can impact the growth and activity of these microbial groups differently, i.e., hydrolytic and acidogenic bacteria generally grow best at a pH of around 6 whereas most methanogens and acetogens have pH optima of around 7 (Lay et al., 1997; Angelidaki et al., 2003, 2011; Pind et al., 2003). Thus, slurry acidification may result in upsetting the anaerobic biodegradation processes and reduce methanogenic activity. In this study we investigated structure and activity responses of bacterial and methanogenic communities to the addition of H_2_SO_4_ to stored liquid dairy manure.

In various manure related environments, culture independent investigations of bacterial and methanogenic communities often involve using phylogenetic and/or functional gene markers (e.g., 16S rRNA and *mcrA* genes) (Petersen et al., 2014; Pandey et al., 2018). However, our previous study indicated that *mcrA* transcripts were more relevant to methane CH_4_ emissions than *mcrA* genes (Habtewold et al., 2018). Particularly with slurry acidification, where significant number of bacterial and methanogenic communities could be dormant or dead, DNA-based studies of these microbes may not reflect activities. Unlike DNA-based studies, changes in the transcriptional levels of phylogenetic and functional marker genes and transcript/functional gene ratios are strong indicators of growth and activity of microbial communities (Freitag and Prosser, 2009; Ma et al., 2012; Blagodatskaya and Kuzyakov, 2013; Wilkins et al., 2015). Hence, in the current study, we aimed to investigate abundance, activity, and diversity responses of bacterial and methanogenic communities in acidified liquid dairy manure by targeting 16S rRNA and *mcrA* genes and transcripts.

## Materials and Methods

### Methane Measurements and Manure Sampling

The study was conducted during the summer season (25 June through 23 October 2017) at the Dalhousie University’s Bio-Environmental Engineering Center (BEEC) in Truro, NS, Canada (45°45′ N, 62°50′ W). Six pilot-scale rectangular outdoor manure storage tanks covered with flow-through steady-state chambers were used. This site has been previously described by Wood et al. (2012). Fresh dairy slurry obtained from a commercial farm was loaded (10.5 m^3^) to each tank. Using duplicate tanks per treatment, 70% H_2_SO_4_ (1.4 L or 2.4 L L^−1^ slurry) or water (2.4 L L^−1^ slurry) were injected (with simultaneous mixing) across the depth of slurries. During storage, gas samples were drawn continuously from the inlet (ambient air) and outlet of each tank using polyethylene tubing, and CH_4_ concentrations were determined at the site using TGA 100A tunable diode laser trace gas analyzer (Campbell Scientific Inc., Logan, UT, United States). Methane flux (g m^−2^ s^−1^) was calculated as described by Wood et al. (2012), and emissions were then converted into daily averages.

For the microbial study, slurry samples were collected before (fresh manure) and after acidification. After acidification, manure samples were collected bi-weekly from the top (10 cm from the surface) and bottom (20 cm from floor) sections of each tank (1.8 cm). From each sampling location, nine slurry samples (on coordinates of a grid) were collected from across the surface and pooled in a clean bucket. Then, two grams subsamples (in duplicate) were collected from each pool in 15 mL Falcon tubes containing 5 mL LifeGuard^TM^ Soil Preservation Solution (MoBio Laboratories Inc., Carlsbad, CA, United States). Samples were then transported to the lab cold and stored in a −20°C freezer until nucleic acid extractions. Based on daily CH_4_ fluxes, manure samples were selected after 20, 50, and 100 days of storage to assess changes in the structure of microbial communities before, during and after peak CH_4_ fluxes, respectively. Sub-samples of appropriate volume were also collected to analyze pH, dry matter (DM), and volatile solid (VS) contents, which were analyzed at the Nova Scotia Department of Agriculture’s Laboratory Services (Harlow Institute, Bible Hill, NS, United States) using standard methods.

### Nucleic Acid Extractions and Quantitative Real-Time PCR

Slurry samples stored with LifeGuard^TM^ Soil Preservation Solution were thawed and centrifuged (4000 × g for 10 min). Pellets were then used to co-extract total RNA and DNA using RNA PowerSoil Total RNA Isolation with DNA Elution Accessory Kits (MoBio Laboratories, Inc., Carlsbad, CA, United States) following the manufacturer’s protocol. Based on information from the manufacturer and our experience, this RNA isolation kit can be used to efficiently isolate RNA and DNA from manure samples as it does for different soil types. As there was little difference in the abundances of bacteria and methanogens between the top and bottom sections of slurries, DNA or RNA samples from these locations were pooled to have one representative sample per tank. RNA samples were reverse transcribed into complementary DNA (cDNA) using Maxima^TM^ H Minus First Strand cDNA Synthesis Kit (Thermo Scientific^TM^) following the manufacturer’s protocol with few modifications. Briefly, 1 μl each of 10× dsDNase Buffer and dsDNase were added to 2 μl (0.3-1 μg) RNA, gently mixed and spun, and incubated at 37°C for 5 min in a preheated thermocycler with lid temperature adjusted to 37°C. After chilled on ice and briefly centrifuged, 4 μl Maxima cDNA H Minus Master Mix (5×) and 6 μl nuclease-free water were added, and gently mixed and centrifuged. For cDNA synthesis reactions, which were performed in a thermocycler with lid temperature adjusted to 50°C, thermal conditions were: 25°C for 10 min, 50°C for 15 min, and 85°C for 5 min. Prior to further downstream analyses, both cDNA and DNA samples were diluted and assessed for potential inhibitory effects as described previously (Habtewold et al., 2017). Diluted DNA (50×) and cDNA (100×) were then used as templates for quantitative real-time polymerase chain reaction (qPCR). Reaction ingredients, conditions, and thermal cycling of qPCR were as described by Habtewold et al. (2017). Known copies of plasmid standard curves for *mcrA* (10e7 to 10e1) and bacterial 16S rRNA (10e9 to 10e1 copies) genes and transcripts were prepared from *Methanosarcina mazei* (ATCC 43340) and a pure culture of *Clostridium thermocellum*, respectively. Efficiency, r^2^, and slope of plasmid standard curve for *mcrA* gene were 98.5 ± 2.8%, 0.99, and −3.34 ± 0.04, whereas for 16S rRNA gene, these values were 98.5 ± 2.7%, 0.99, and −3.36 ± 0.07, respectively. CFX Manager software version 3.1 (Bio-Rad Laboratories, Inc., Hercules, CA, United States) and GraphPad prism v.7 (GraphPad Software, Inc) were used to analyze the qPCR data.

### Amplicon Library Preparation and Sequencing

Methane fluxes from all acidified slurries were very low, thus slurries treated with 2.4 L 70% H_2_SO_4_ m^−3^ slurry (acidified slurries) and untreated slurries were selected to study the effects of acidification on community structure of bacteria and methanogens. Polymerase chain reaction (PCR) primers (515FB-806RB) that target the V4 region of bacterial and archaeal 16S rRNA genes were used to prepare 16S rRNA gene and transcript libraries (Walters et al., 2016). To study methanogens, the gene encoding the alpha subunit of methyl coenzyme M reductase (*mcrA*) which is a key enzyme in methanogenesis was targeted using mlas-mod and mcrA-rev primers (Angel et al., 2011). On both 16S rRNA and *mcrA* gene primers, Illumina adapter sequences A and B (Supplementary Table S1) were added to the 5′-ends of the forward and reverse primers, respectively. For both genes, amplicons were prepared in two PCR steps with a total of 35 cycles. First, duplicate 25 μL PCR reactions per sample were prepared by adding 5 μL of 5X Phusion HF buffer, 0.25 μL of Thermo Scientific^TM^ Phusion^TM^ Hot Start II High-Fidelity DNA Polymerase (Thermo Scientific), 0.5 μL of 10 mM dNTPs (Thermo Scientific), 0.5 μL of each of the forward and reverse primers (10 μM), 2 μL of diluted DNA (10–50 ng/μL) or cDNA, and 16.25 μL nuclease free water. Thermal cycling for both genes were as follows: initial denaturation at 98°C for 3 min, followed by 25 cycles of dissociation at 98°C for 10 s, primer annealing (50°C and 55°C for 30 s, for 16S rRNA and mcrA gene/transcript, respectively), extension at 72°C for 30 s, and a final extension for 10 min. Duplicate PCR reactions were pooled and products were cleaned using silica spin columns (Wizard^®^ SV Gel and PCR Clean-Up System; Promega) following the recommended protocol. The second step PCR was performed for 10 cycles to attach Illumina index tags to the ends of the amplicons that were obtained from the first-step PCR. For each sample, a different combination of the Index primers 1 (N7xx) and Index primers 2 (S5xx) of Illumina’s Nextera^®^ XT DNA Library Preparation Kit (Illumina Inc., San Diego, CA, United States) were used to perform PCR. This was performed in a single 50 μL reaction mix per sample, and same proportion of reagents and thermal cycling conditions were used as the first-step PCR except the 4 μL purified amplicons template DNA. PCR products were then purified by magnetic beads (Agencourt AmPure XP; Beckman Coulter, Brea, CA, United States) and re-suspended in 25 μL. Purified PCR products were tested for correct amplicon length using gel electrophoresis and submitted to the University of Guelph Advanced Analysis Centre, Genomic Facility (Guelph, ON, Canada) for sequencing. Prior to sequencing, libraries were normalized by Sequalprep (Thermo Fisher Scientific, Hampton, NH, United States) and library quality was assessed from 6 randomly selected samples using Bioanalyzer DNA1000 chip (Agilent, Santa Clara, CA, United States). Multiplexed sample sequencing was conducted using MiSeq v3 600 cycle reagent kit (Illumina Inc., San Diego, CA, United States) producing 2 × 300 bp. Unprocessed FASTQ files were received for subsequent analysis.

### Sequence Data Analysis

Raw sequence data of 16S rRNA genes and transcripts were processed and analyzed in Mothur v.1.39.5 (Schloss et al., 2009) following the recommended pipeline (Kozich et al., 2013). Briefly, forward and reverse reads of each sample were merged, target-specific primer sequences removed, and sequences were screened for ambiguity and length. Then, sequences were aligned against the Silva reference sequence (release 132), further screened for length and homopolymer, overhangs and common gaps filtered, and pre-clustered to further denoise sequencing errors. After removal of potential chimeric sequences, Mothur-formatted version of the RDP’s 16S rRNA reference (version 16) was used to classify sequences into phylotypes at 80% cut-off in which undesirable targets that might have been picked by primers were filtered. Finally, purified sequences were clustered into operational taxonomic units (OTUs) at 0.03 cut-off (97% similarity), and phylotypes of OTUs identified using the RDP’s 16S rRNA reference database. The *mcrA* gene and transcript sequences were processed similarly except that non-target reads and potential frameshift errors were removed or corrected using the FrameBot function of the RDP’s Functional Gene and Repository Pipeline tool (Fish et al., 2013; Wang et al., 2013). OTU-based alpha diversity (e.g., rarefaction, coverage, Chao1, and Inverse Simpson diversity estimate) and beta diversity (e.g., non-metric multidimensional scaling) analyses were performed in Mothur. Significance of differences in diversity, richness, and composition microbial community between treatments were tested in Mothur, STAMP (statistical analysis of taxonomic and functional profiles), and GraphPad prism v.7 GraphPad Software, Inc.) (Schloss et al., 2009; Parks et al., 2014).

### Sequence Accessions

Raw reads of 16S rRNA and *mcrA* genes and transcripts have been deposited in NCBI’s short read archives as FASTQ files under the accession number SRP119447.

## Results

### Manure Characteristics, CH_4_ Flux and Microbial Abundance

**FIGURE 1:**
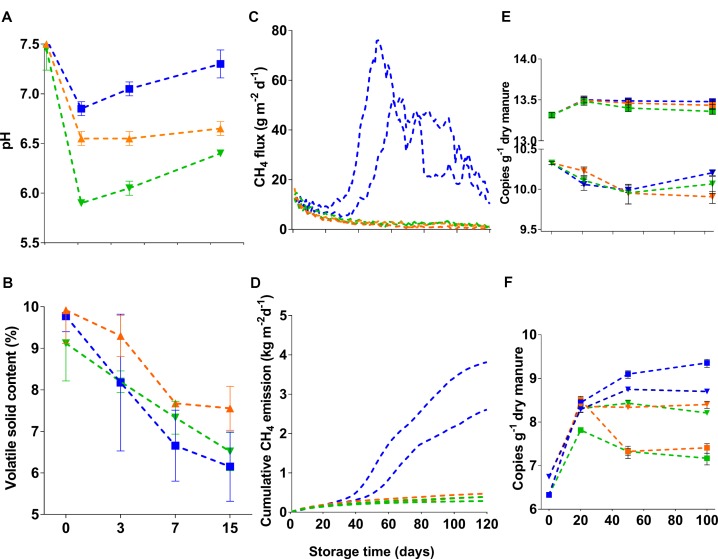
Manure characteristics (**A**: pH; **B**: volatile solid content), CH_4_ emissions (**C**: daily flux; **D**: cumulative emissions), and abundance of microbial communities (**E**: bacteria: **F**: methanogens). In **(E,F)**, filled down triangle and square symbols indicate gene and transcript copies, respectively. The blue color in all figures indicates untreated manure whereas the yellow-brown and green colors indicate manures that received 1.4 L and 2.4 L 70% H_2_SO_4_, respectively.

Initially, the pH of fresh dairy manure used in the current study was 7.5. Twenty days after addition (and mixing) of 1.4 L or 2.4 L 70% H_2_SO_4_ per cubic meter of dairy slurry, mean pHs of slurries were 6.5 ± 0.1 and 5.9 ± 0.01, respectively (Figure 1A), while the pH of untreated slurries was 6.8 ± 0.07. After 50 days of storage, slurry pH gradually increased in all tanks by 0.35 ± 0.2. Nevertheless, pH increases in acidified slurries were small when compared with untreated control. Regardless of treatments, VS contents of slurries declined during storage (Figure 1B). Total and ammoniacal nitrogen contents (in %) of the fresh manure were 0.46 ± 0.01 and 0.18 ± 0.01, respectively. At the end of the storage period, these values were reduced to 0.37 ± 0.07 and 0.16 ± 0.02, and 0.42 ± 0.06 and 0.16 ± 0.03, in the untreated and acidified slurries, respectively. Unlike the untreated slurries, where peak CH_4_ fluxes (76–52 g m^−2^ d^−1^) were detected between 50 and 60 days of storage (Figure 1C), fluxes from the acidified slurries were consistently low (<10 g m^−2^ d^−1^) throughout the storage period. Addition of 1.4 L and 2.4 L 70% H_2_SO_4_ m^−3^ slurry resulted in 69–84% reduction of cumulative CH_4_ emissions when compared with untreated slurries (Figure 1D).

Fresh manure (<1-day old) had a large number of bacteria, where copy numbers (Log10) of 16S rRNA genes and transcripts were 10.3 and 13.3 g^−1^ dry manure, respectively (Figure 1E). After 20 days of storage, the abundance of bacteria decreased in both acidified and untreated slurries. However, there were no significant differences (Kruskal–Wallis followed by Dunn’s test) in the abundance of bacteria between acidified and untreated slurries (Figure 1E). For instance, when CH_4_ flux peaked in untreated slurries (after 50 days of storage), differences in the copy numbers of 16S rRNA genes in acidified and untreated slurries were only about 0.4%. These small differences slightly increased (up to ∼3%) after 100 days of storage, but were not statistically significant. Thus, storage time had a greater impact on bacterial abundance than slurry acidification. Similarly, the activity of bacteria (as estimated from 16S rRNA transcript copies g^−1^ dry manure) showed little variation (up to 1%) with slurry acidification (Figure 1E). These results indicated that neither abundance nor activity were altered with manure acidification (pH up to 5.9).

Unlike bacteria, slurry acidification negatively affected the abundance and activity methanogenic populations. Fresh manure had 6.74 ± 0.05 copies (Log10) of *mcrA* genes g^−1^ dry manure (Figure 1F). After 20 days of storage, the *mcrA* gene copies showed significant increases (∼30% and ∼25% in untreated and acidified slurries, respectively; Dunn’s test, *p* < 0.0001). The effect of slurry acidification however was not noticeable after 20 days of storage (Figure 1F). After 50 days of storage, there were significantly lower numbers (4–4.7%; Dunn’s test, *p* = 0.047) of *mcrA* gene copies (Log10 transformed) in acidified slurries. These differences slightly increased (5–6%; Dunn’s test, *p* = 0.006) after 100 days of storage. The effect of slurry acidification on the activity of methanogens (estimated from *mcrA* transcript copies g^−1^ dry manure) was more significant. After 20 days of storage, slurries that received ∼2.4 L 70% H_2_SO_4_ m^−3^ slurry showed ∼6.5% lower copies of *mcrA* transcripts, while *mcrA* transcript/gene ratios were reduced by ∼1.7% (Supplementary Figure S2). Significant reductions (up to 32%; Dunn’s test, *p* < 0.0001) of *mcrA* transcript copies (Log10) were detected after 50 and 100 days of storage. During these time periods, *mcrA* transcript/gene ratios in acidified slurries were also reduced by ∼21 and 25%, respectively (Supplementary Figure S2).

### Effects of Manure Acidification on the Diversity of Bacteria and Methanogens

After quality inspections of raw MiSeq sequencing data, 1863088 quality reads of 16S rRNA gene and transcript (an average of 71657 per sample) were obtained. Similarly, 524245 quality reads of *mcrA* gene and transcript (an average of 20163 per sample) were obtained. Diversity and community composition of bacteria and methanogens were analyzed after singletons were removed from 16S rRNA and *mcrA* genes and transcripts reads (Auer et al., 2017). Rarefaction plots for both gene and transcript indicated sufficient sampling efforts that might have covered most bacterial and methanogenic communities in the manure (Supplementary Figures S1a,b).

Shifts in the diversity of bacteria and methanogens due to acidification and/or storage time were shown using Inverse Simpson diversity index. Bacterial and methanogen diversity in 16S rRNA and *mcrA* gene libraries were higher at the beginning of storage (Table 1). However, diversities in the corresponding transcript libraries were reduced by half. These differences were consistent throughout the storage period, which might indicate the inability of several bacterial taxa from fresh manure to adapt to storage conditions. Unlike bacteria, there were significant differences (*t*-test, *p* < 0.05) in the diversity of methanogens between acidified and untreated slurries (Table 1). Diversity in *mcrA* gene and transcript libraries of untreated slurries also increased with storage period. These results indicated that manure acidification results in stronger impacts on methanogens when compared with bacteria.

**Table 1:** Richness and diversity analysis of bacteria and methanogens from dairy manure.

Days	Treatment	S_obs_	S_chao_	Invsimpson	S_obs_	S_chao_	Invsimpson
		
		16S rRNA gene			*mcrA* gene		
0		2951	3653	50	49	49	2.93
20	Control	3052 ± 192	4383 ± 99	40 ± 3	41 ± 3	54 ± 10	1.41 ± 0.09
50		2955 ± 83	4571 ± 23	45 ± 1	47 ± 5	57 ± 11	1.8 ± 0.15
100		2910 ± 13	3997 ± 216	42 ± 7	43 ± 3	48 ± 4	2.38 ± 0.3
20	Acidified	3088 ± 111	4651 ± 42	38 ± 1	45 ± 3	65 ± 11	1.64 ± 0.03
50		3205 ± 150	4670 ± 204	41 ± 6	39 ± 1	73 ± 48	1.39 ± 0.08
100		2721 ± 510	3861 ± 536	39 ± 26	42 ± 1	51 ± 6	1.33 ± 0.03

		**16S rRNA transcript**			***mcrA* transcript**		
**0**		**1985**	**3044**	**27**	**16**	**23**	**1.49**

20	Control	3030 ± 223	4506 ± 157	26 ± 5	19 ± 1	20 ± 2	1.39 ± 0.4
50		2890 ± 695	4385 ± 723	22 ± 3	17 ± 1	17 ± 1	2.06 ± 0.03
100		2938 ± 397	4250 ± 542	22 ± 4	25 ± 2	36 ± 1	2.51 ± 0.1
20	Acidified	3113 ± 424	4596 ± 321	19 ± 1	15 ± 2	17 ± 3	1.38 ± 0.24
50		2739 ± 806	4407 ± 836	19 ± 2	10 ± 1	16 ± 6	1.37 ± 0.28
100		3058 ± 113	4474 ± 357	22 ± 9	8 ± 2	11 ± 7	1.45 ± 0.08

*Mean and standard deviation of biological replicates (n = 2) are shown in the table. S_obs_, S_chao_, and Invsimpson, indicate Species observed, Species estimated, and Inverse Simpson index, respectively.*

**FIGURE 2:**
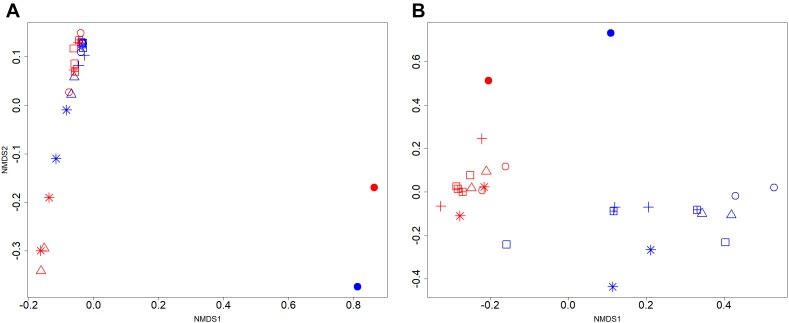
Two-dimensional non-metric multidimensional scaling (NMDS) of **(A)** bacterial and **(B)** methanogenic communities in stored liquid dairy manure. Samples from day 0, 20, 50, and 100 of untreated slurries were indicated by the filled circle, open circle, open triangle, and asterisk, respectively. Acidified slurries from day 20, 50, and 100 were indicated by the plus, square plus, and open square, respectively. Blue and red colors indicated gene and transcripts libraries, respectively.

The effects of manure acidification on the community structure of bacteria and methanogens were indicated by NMDS plots (Figures 2A,B). With a good fit of ordination (2D Stress = 0.09; *r*^2^ = 0.97), the NMDS plot did not show distinct clustering of bacterial communities from acidified and untreated slurries (Figure 2A). In line with the Inverse Simpson diversity estimates, bacterial communities in 16S rRNA gene and transcript libraries showed significant separation (AMOVA, *p* < 0.001) regardless of treatments. Methanogens from acidified and untreated slurries particularly after 50 days of storage showed distinct NMDS clustering patterns (2D Stress = 0.07; *r*^2^ = 0.99) which supported the diversity estimates. While the methanogens in *mcrA* transcript libraries of untreated slurries clustered separately from those in *mcrA* gene libraries (AMOVA, *p* < 0.05), no significant separation was observed for the acidified slurries (Figure 2B).

### Effects of Acidification on Relative Proportions of Bacterial and Methanogenic Phylotypes

**FIGURE 3:**
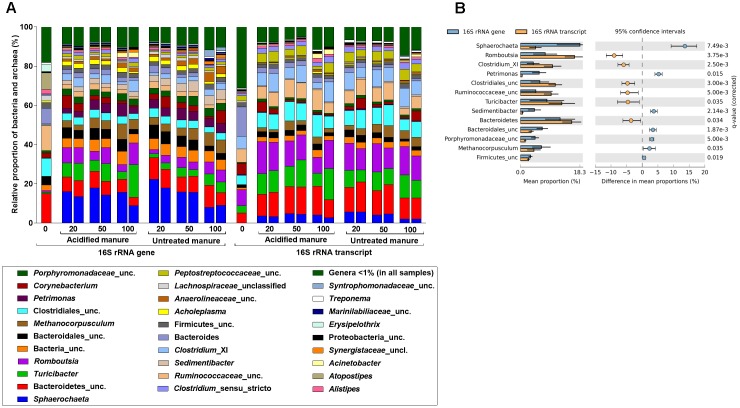
Effects of manure acidification on bacterial and archaeal phylotypes as indicated by **(A)** relative proportions of abundant genera in fresh, acidified and untreated slurries **(B)** extended error bar plots illustrating significantly abundant bacteria (Effect size = 2, White’s non-parametric *t*-test with Benjamini–Hochberg multiple test correction, *q*-values < 0.05) in acidified and untreated slurries. Numbers on the X axis indicate storage time in days. Except fresh manure, all have biological replicates (*n* = 2).

In this study, 95.5% of the 16S rRNA gene and transcript reads were related to bacteria (Supplementary Figure S3a). In fresh manure, phylum Firmicutes and Bacteroidetes predominated (55–57% and 32–24%, respectively) 16S rRNA gene and transcript libraries (Supplementary Figure S3b). Relative proportions of the predominant bacterial phyla did not vary with slurry acidification. Regardless of treatments, 16S rRNA gene libraries from stored slurries were dominated by Firmicutes (35 ± 8%), Bacteroidetes (25 ± 4%), and Spirochaetes (15 ± 4%) whereas Firmicutes (59 ± 5%) and Bacteroidetes (21 ± 3%) predominated the 16S rRNA transcript libraries (Supplementary Figure S3b). At the genus level, *Sphaerochaeta* was the most abundant (15 ± 4%) bacteria in 16S rRNA gene libraries (Figure 3A). Uncultured members of Bacteroidetes (8 ± 2%), *Turicibacter* (6 ± 4%), and *Romboutsia* (6 ± 3%) were also predominant in both acidified and untreated slurries. In 16S rRNA transcript libraries of both acidified and untreated slurries, many members of Firmicutes (e.g., *Romboutsia*, *Turicibacter*, uncultured Clostridiales, *Clostridium*_XI, uncultured *Ruminococcaceae*) and uncultured Bacteroidetes accounted for 46 ± 4% and 13 ± 2%, respectively (Figure 3A).

Analysis of differences in mean proportions of 16S rRNA gene and transcript reads between acidified and untreated slurries (White’s non-parametric *t*-test with Bonferroni correction, CI = 95%, *α* = 0.05) indicated that manure acidification with H_2_SO_4_ did not alter the composition of bacterial communities. Regardless of treatments, 16S rRNA gene and transcript communities were significantly different (White’s non-parametric *t*-test, *p* = 0.015; Figure 3B), which was in line with the NMDS analysis. Genera from different bacterial phyla (e.g., *Spirochaeta*, *Petrimonas*, and *Sedimentibacter*) and members of the Firmicutes (e.g., *Romboutsia*, *Clostridium*_XI, and uncultured members *Ruminococcaceae*) were represented differently in the 16S rRNA gene and transcript communities.

**FIGURE 4:**
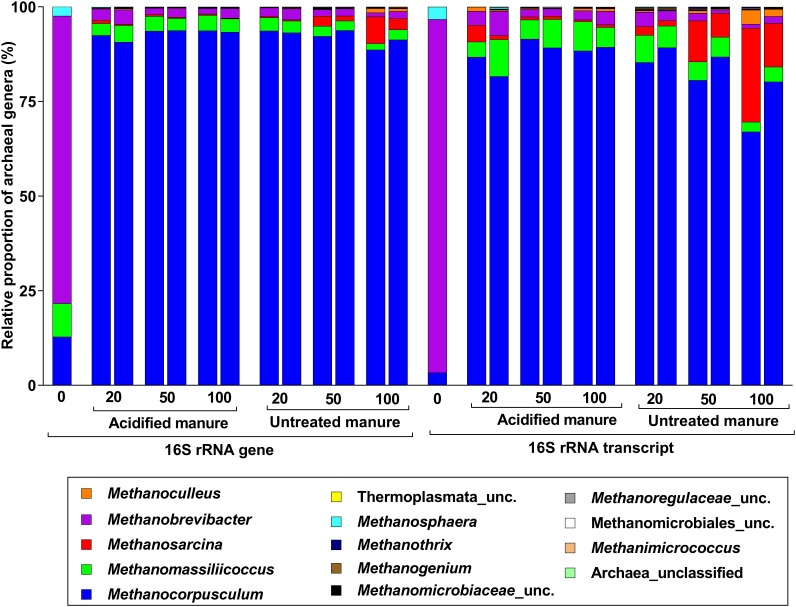
Taxonomic distribution of archaeal phylotypes identified from 16S rRNA gene and transcript reads. Numbers on the X-axis show storage time in days. Except fresh manure, all have biological replicates (*n* = 2).

Archaea accounted for 4.5% of 16S rRNA gene and transcript reads (Supplementary Figure S3a), and all were methanogens. The most abundant genus in fresh manure was *Methanobrevibacter* (76%), but *Methanocorpusculum* was predominant (92 ± 1% and 86 ± 2% in archaeal 16S rRNA gene and transcript libraries, respectively) in stored slurries of all treatments (Figure 4). While the proportion of *Methanosarcina* in 16S rRNA gene and transcript libraries of untreated slurries gradually increased (up to 5 and 18%, respectively), its proportions in acidified slurries was consistently below 1%. Thus, *Methanosarcina* seemed strongly inhibited by manure acidification.

**FIGURE 5:**
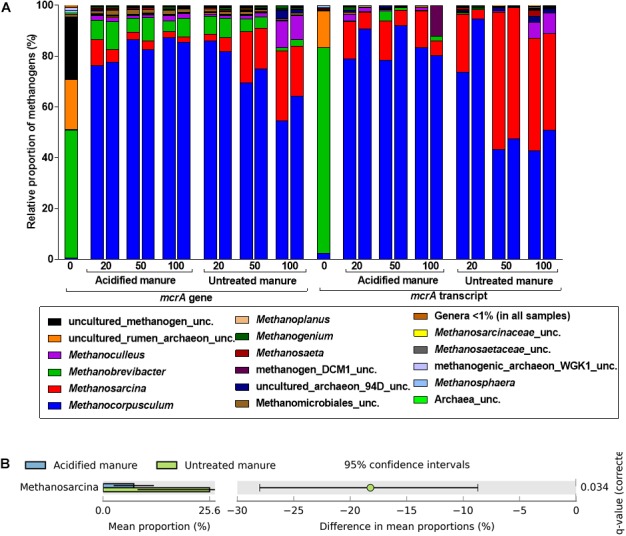
Effects of manure acidification on methanogens as indicated by **(A)** a stacked bar showing relative proportions of methanogenic genera in fresh, acidified and untreated slurries **(B)** extended error bar plots illustrating significantly abundant methanogens (Effect size = 2, White’s non-parametric *t*-test with Benjamini–Hochberg multiple test correction, *q*-values < 0.05) in acidified and untreated slurries. Numbers on the *X* axis indicate storage time in days. Except fresh manure, all have biological replicates (*n* = 2).

Like in archaeal 16S rRNA gene libraries, *Methanobrevibacter* predominated the *mcrA* gene library from fresh manure (Figure 5A). Although *Methanocorpusculum* represented < 1% of the methanogens in fresh manure, it represented 83 ± 3% of the *mcrA* gene and transcript reads from acidified slurries. *Methanocorpusculum* was also dominant in untreated slurries but gradually declined (84 to 59% and 84 to 46% in the *mcrA* gene and transcript libraries, respectively) with storage time. In contrast, the relative proportion of *Methanosarcina*, increased (4 to 25% and 13 to 41% in the *mcrA* gene and transcript libraries, respectively) in untreated slurries, but in acidified slurries the population remained stable. Increased amounts of CH_4_ emitted from untreated slurries coincided with the increased abundance of *Methanosarcina*.

Analysis of differences in mean proportions of *mcrA* gene reads between acidified and untreated slurries indicated that *Methanosarcina* (*p* = 0.05) was differentially enriched in untreated slurries (Figure 5B), indicating the negative impacts of manure acidification on methanogens related to the genus *Methanosarcina*.

## Discussion

Stored liquid dairy manure is a point source of CH_4_. Studies have demonstrated that H_2_SO_4_-based acidification of liquid dairy manure (reducing pH to ∼5.5) can reduce CH_4_ emissions from 67 to 90% (Petersen et al., 2012, 2014; Misselbrook et al., 2016; Sommer et al., 2017). In line with these studies, 69–84% reductions of cumulative CH_4_ emissions were detected in this study after acidification of dairy manure to pH 6.5 and 5.9. Thus, small changes in slurry pH were able to disrupt methanogenesis that has pH optima around 7 (Lay et al., 1997; Liu et al., 2008; Weiland, 2010; Mao et al., 2017); however, reduction in CH_4_ emissions could also be related to the toxicity of hydrogen sulfide that could be accumulated as a result of potential sulfate reduction (Petersen et al., 2012). Throughout the storage period, CH_4_ fluxes from acidified slurries remained low, consistent with levels observed during the lag phase (the first 20 days of storage, Figure 1C). The lag phase observed in the current study was also observed in our previous studies that did not involve acidification (Habtewold et al., 2018). A previous study from our lab has linked shifts in methanogens to methane emissions (Habtewold et al., 2018), therefore we predicted that the communities would shift after acidification. Using qPCR-based quantification and deep sequencing (Illumina MiSeq) of phylogenetic and functional marker genes and transcripts, we demonstrated that slurry acidification (to pH 5.9) did not affect the community structure of most anaerobically-degrading microorganisms except methanogens closely related to the genus *Methanosarcina*. As major players in CH_4_ production (Conrad, 1999), impacts on methanogens related to *Methanosarcina* can have a drastic effect on methanogenesis and CH_4_ emissions.

With slurry acidification (mean pHs 6.5 and 5.9), shifts in the abundance and activities of bacterial communities, as estimated from 16S rRNA gene and transcript copies, were not significant. Thus, acidification might have little effect on growth and activities of bacterial communities involved in the anaerobic degradation of organic matter (e.g., hydrolytic, acidogenic, and acetogenic bacteria) in manure (Lin et al., 2013; Kuruti et al., 2017). As acidification may reduce aggregation of slurry particles (Fangueiro et al., 2015; Gomez-Munoz et al., 2016; Regueiro et al., 2016), substrate availability for hydrolytic and acidogenic bacteria in slurries may increase. Gradual reductions in the volatile solids contents of both acidified and untreated slurries indicated that these communities were active. Microbial consumption of volatile solids in manure typically increases the amount of organic acids (e.g., acetic, propionic, and butyric acids) and methanogenic substrates (e.g., CO_2_, H_2_, acetate, formate, and alcohol). Thus, pH reductions observed during the first 20 days of storage (regardless of treatment) might be related to accumulation of organic acids. Although pH reductions due to organic acid accumulation might be obscured in acidified slurries, reductions in total solids contents in these slurries might indicate microbial activities. Regardless of treatments, slurry pH gradually increased after 50 days of storage which was in line with other studies (Patni and Jui, 1985; Sommer et al., 2017). However, pH increases (often due to consumption of organic acids by acetogens and methanogens) in acidified slurries were lower when compared with untreated slurries. These small changes in pH coincided with low CH_4_ flux from acidified slurries, indicating negative impacts of acidification with H_2_SO_4_ on methanogens.

Acidification had little effect on the abundance of methanogenic populations. This was in line with a study by Petersen et al. (2014) where the abundance of methanogens in pig slurry did not shift with slurry acidification (pH down to 5.5). However, the authors detected more than 90% reductions in CH_4_ emission which indicate the negative impacts of slurry acidifications with H_2_SO_4_ on methanogenic processes. Ottosen et al. (2009) also detected significant reductions (>98%) in microbial processes (oxygen consumption rate, methanogenesis and sulfate reduction) in acidified pig slurry. As DNA-based studies of *mcrA* genes provide information about all methanogens (active, dormant, and dead), in this study we used instead mRNA of the *mcrA* genes (*mcrA* transcript) to specifically study changes in physiological status of methanogens and methanogenic processes. Unlike population abundance, the reduced copy numbers of *mcrA* transcripts in acidified slurries with negligible CH_4_ emissions might reflect the negative effect of manure acidification on the activities of methanogens. However, some or most methanogens might still grow and function in acidified slurries as the abundance and activities of methanogens in it were higher when compared with fresh manure. This would account for the residual methane emission observed.

With little impacts of acidification on the abundance and activity of bacteria, accumulated intermediary compounds including propionate, butyrate, and valerate could be converted into acetate by the acetogens (Demirel and Scherer, 2008), making stored liquid dairy manure rich in acetate (Barret et al., 2013; Habtewold et al., 2018). Although acetoclastic methanogenesis (using acetate as substrate) is the major contributor of CH_4_ produced in many environments (Conrad, 1999), CH_4_ production in environments with high concentration of acetate has been found to drastically reduce as pH decline (Van Kessel and Russell, 1996), although the exact mechanism is not yet clear.

Consistent with the qPCR data, the diversity and relative proportions of bacterial communities were not altered with slurry acidification (to pH 5.9). Regardless of treatments, *Sphaerochaeta* was predominant in the 16S rRNA gene libraries. These bacteria are enriched with fermentation and carbohydrate metabolism genes (Caro-Quintero et al., 2012), but it is unclear why they represented lower proportions in the 16S rRNA transcript libraries where several fermentative bacteria (e.g., *Turicibacter*, *Bacteriodetes*, and *Romboutsia*) (Bosshard et al., 2002; Thomas et al., 2011; Gerritsen et al., 2017) were predominant. The abundance of these bacteria, particularly in the 16S rRNA transcript libraries of acidified slurries, might indicate availability of methanogenic substrates. Regardless of treatments, methanogens closely related to the genus *Methanocorpusculum* that are known to perform hydrogenotrophic methanogenesis (reducing CO_2_ to CH_4_ using hydrogen) predominated the archaeal 16S rRNA and *mcrA* gene and transcript libraries. This was in line with our previous pilot-scale studies conducted using manure imported from the same commercial farm (Habtewold et al., 2017, 2018). However, CH_4_ emissions were significant only in untreated slurries where the proportion of methanogens closely related to the genus *Methanosarcina* had significantly increased. In contrast to many other methanogens, *Methanosarcina* has been reported to grow under high concentrations of ammonia and VFA (Demirel and Scherer, 2008). However, the current study indicated that these methanogens were apparently impacted by the acidification with H_2_SO_4_ and perhaps by products of sulfate reduction (e.g., H_2_S). Compared to acidified slurries, the predominance of *Methanosarcina* was high in untreated slurries which coincided with increased CH_4_ emissions. *Methanosarcina* species are metabolically the most diverse and have higher efficiency in CH_4_ production (e.g., 3× when glucose is used as substrate) when compared with *Methanocorpusculum* (Conrad, 1999; Kotsyurbenko et al., 2004), thus any effect on these methanogens might result in significant reduction of CH_4_ production.

In stored liquid manure, reductions in CH_4_ emissions might also be related to potential methanotrophy, which is presumed to occur in the surface crusts of slurries where oxygen is freely available for methanotrophs (Petersen and Ambus, 2006). In the current study, no crust was formed, and no known methanotroph was detected in both the 16S rRNA gene and transcript libraries of all treatments. Thus, the contribution of methanotrophy to the reduction of CH_4_ emissions detected in the current study were less likely.

With the use of H_2_SO_4_ for manure acidification, slurries can be enriched with sulfate which is an important substrate for sulfate-reducing bacteria that have high affinity to available hydrogen (Kristjansson and Schönheit, 1983). Although the relative proportions of sulfate-reducers detected in the current study (e.g., *Desulfatibacillum*, *Desulforhopalus*, *Desulfuromonas*, and *Desulfobulbus*) were low, together with potential homoacetogenic bacteria (e.g., *Acetobacterium* and *Blautia*), they might still compete hydrogenotrophic methanogens for available substrates (Weijma et al., 2002). Methanogens related to the genus *Methanosarcina* can perform all three pathways of methanogenesis (hydrogenotrophic, acetoclastic, and methylotrophic), thus may compete favorably by changing substrates. Although sulfate-reducing bacteria can also compete for acetate, this substrate is highly abundant in stored liquid dairy manure (Barret et al., 2013; Habtewold et al., 2018), and acetate consumption rates in methanogens are relatively higher when compared to sulfate reducers (Bhattacharya et al., 1996). Hydrogen sulfide, which could be accumulated in slurries as a result potential sulfate reduction, might also suppress the activities of methanogens except *Methanosarcina* (Demirel and Scherer, 2008). Thus, differential enrichment of *Methanosarcina* in untreated slurries indicated manure acidification with H_2_SO_4_ had more impact on these methanogens when compared to *Methanocorpusculum*.

## Conclusion

H_2_SO_4_-based acidification of stored liquid dairy manure (mean pH 6.5 and 5.9) could reduce cumulative CH_4_ emissions by 76 ± 7% and 78 ± 6%, respectively. Slurry acidification (pH down to 5.9) with H_2_SO_4_ coincided with significant reduction of VS contents of slurries in all treatments, but did not significantly impact the abundance, activity or community structure of bacteria. Regardless of treatments, *Methanocorpusculum* was the predominant methanogenic genus. *Methanosarcina*, while representing a minor proportion of the methanogens in this dairy slurry, was relatively lower in acidified slurries, and this coincided with significant reductions in CH_4_ emissions. Thus, we propose that manure acidification with H_2_SO_4_ reduced CH_4_ emissions by inhibiting growth and activities of *Methanosarcina*, the most metabolically diverse methanogen.

## Author Contributions

All the authors were involved in the planning of the work and revision of the manuscript. JH conducted the molecular work, data analysis, interpretations, and prepared the manuscript with the guidance of KD and RG.

## Conflict of Interest Statement

The authors declare that the research was conducted in the absence of any commercial or financial relationships that could be construed as a potential conflict of interest.
