# Supplementary material for: Reduction in Methane Emissions From Acidified Dairy Slurry Is Related to Inhibition of Methanosarcina Species
Source: Front Microbiol. 2018 Nov 20;9:2806. doi: 10.3389/fmicb.2018.02806 (PMC6255968; doi:10.3389/fmicb.2018.02806)
Supplement: Supplementary file 1 [file Data_Sheet_1.docx]

Supplementary Material

Reduction in Methane Emissions from Acidified Dairy Slurry is Related to Inhibition of *Methanosarcina* Species

**Jemaneh Habtewold^a^, Robert Gordon^b^, Vera Sokolov^b^, Andrew VanderZaag^c^, Claudia Wagner-Riddle^a^, Kari Dunfield*^a^**

*** Corresponding author:**

Dr. Kari Dunfield

School of Environmental Sciences, University of Guelph

Guelph, N1G 2W1, ON, Canada

[dunfield@uoguelph.ca](mailto:dunfield@uoguelph.ca)

# Supplementary Tables

**Table S1.** PCR primers used, and Illumina adaptors used in this study.

| Name | Sequence (5’-3’) | Use | Reference |
| --- | --- | --- | --- |
| mlas-mod F | GGYGGTGTMGGDTTCACMCARTA | qPCR (methanogens) | (Angel et al., 2011) |
| mcrA-rev | CGTTCATBGCGTAGTTVGGRTAGT |  |  |
| Bac338F | ACTCCTACGGGAGGCAGCAG | qPCR (bacteria) | (Fierer et al., 2005) |
| Bac518R | ATTACCGCGGCTGCTGG |  |  |
| 515FB | GTGYCAGCMGCCGCGGTAA | Amplicon preparation (bacteria) | (Walters et al., 2016) |
| 80bRB | GGACTACNVGGGTWTCTAAT |  |  |
| Adaptor A | GTCTCGTGGGCTCGGAGATGTGTATAAGAGACAG | Illumina MiSeq sequencing | |
| Adaptor B | TCGTCGGCAGCGTCAGATGTGTATAAGAGACAG |  |  |

# Supplementary Figures

#
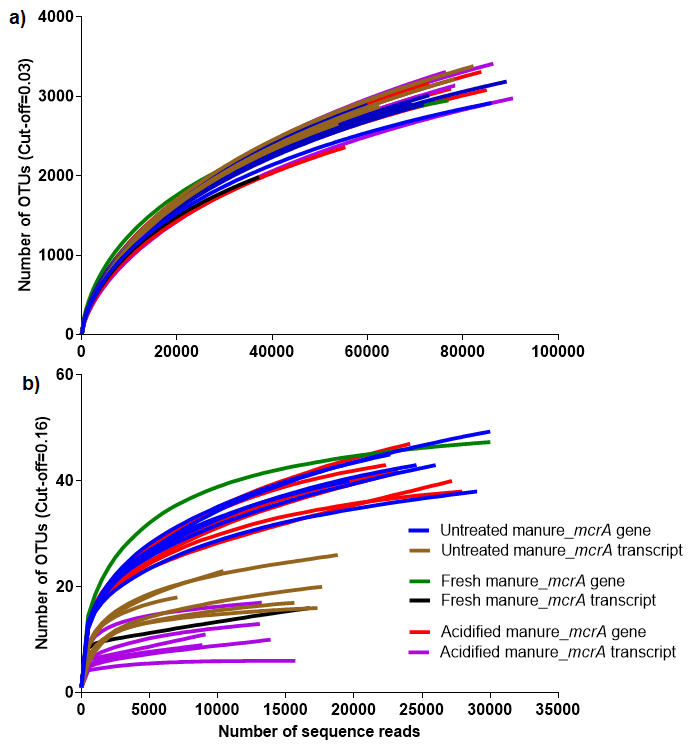
Figure S1. Rarefaction curves, as calculated in Mothur, showing changes in the number of observed OTUs of a) bacteria and archaea (16S rRNA sequence reads, cut-off=0.03) and b) methanogens (*mcrA* sequence reads, cut-off=0.16) as sampling intensity (number of sequences sampled) increases from dairy manure.

#

# Figure S2. *mcrA* transcript: gene ratios in stored liquid dairy manure.

#
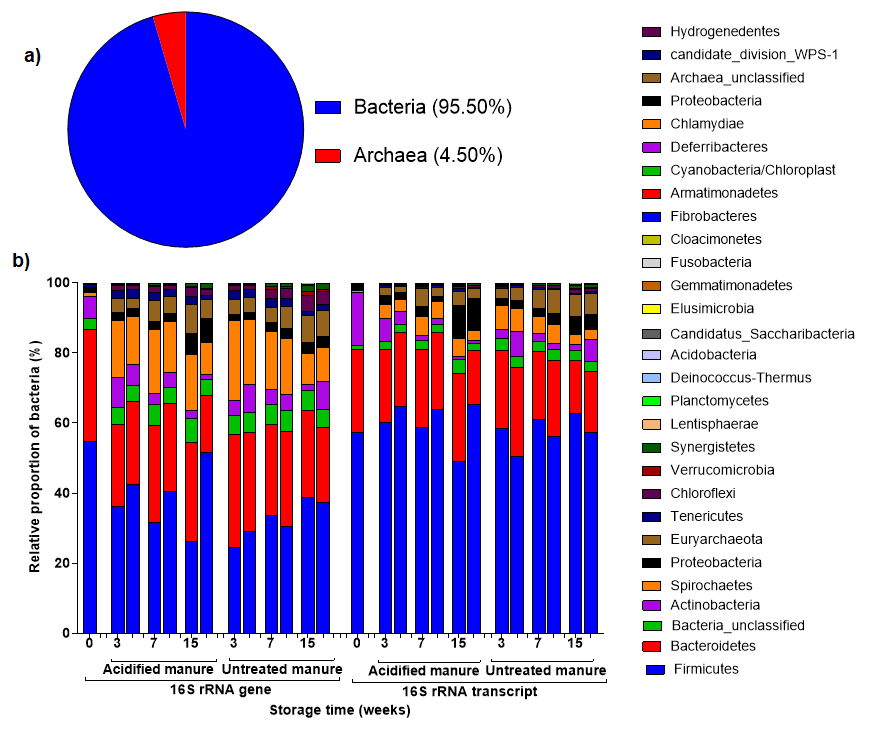


# Figure S3. a) overall proportion of bacteria and archaea in 16S rRNA gene and transcript libraries b) taxonomic distribution of 16S rRNA gene and transcript phylotypes at phylum level.

**References**

Angel, R., Matthies, D., and Conrad, R. (2011). Activation of methanogenesis in arid biological soil crusts despite the presence of oxygen. *PLoS One* 6(5)**,** e20453. doi: 10.1371/journal.pone.0020453.

Fierer, N., Jackson, J.A., Vilgalys, R., and Jackson, R.B. (2005). Assessment of soil microbial community structure by use of taxon-specific quantitative PCR assays. *Applied and environmental microbiology* 71(7)**,** 4117-4120.

Walters, W., Hyde, E.R., Berg-Lyons, D., Ackermann, G., Humphrey, G., Parada, A., et al. (2016). Improved Bacterial 16S rRNA Gene (V4 and V4-5) and Fungal Internal Transcribed Spacer Marker Gene Primers for Microbial Community Surveys. *mSystems* 1(1). doi: 10.1128/mSystems.00009-15.
